# Personal exposure to fine particulate air pollution while commuting: An examination of six transport modes on an urban arterial roadway

**DOI:** 10.1371/journal.pone.0188053

**Published:** 2017-11-09

**Authors:** Robert A. Chaney, Chantel D. Sloan, Victoria C. Cooper, Daniel R. Robinson, Nathan R. Hendrickson, Tyler A. McCord, James D. Johnston

**Affiliations:** Brigham Young University, Department of Health Science, Provo, Utah, United States of America; Utah State University, UNITED STATES

## Abstract

Traffic-related air pollution in urban areas contributes significantly to commuters’ daily PM_2.5_ exposures, but varies widely depending on mode of commuting. To date, studies show conflicting results for PM_2.5_ exposures based on mode of commuting, and few studies compare multiple modes of transportation simultaneously along a common route, making inter-modal comparisons difficult. In this study, we examined breathing zone PM_2.5_ exposures for six different modes of commuting (bicycle, walking, driving with windows open and closed, bus, and light-rail train) simultaneously on a single 2.7 km (1.68 mile) arterial urban route in Salt Lake City, Utah (USA) during peak “rush hour” times. Using previously published minute ventilation rates, we estimated the inhaled dose and exposure rate for each mode of commuting. Mean PM_2.5_ concentrations ranged from 5.20 μg/m^3^ for driving with windows closed to 15.21 μg/m^3^ for driving with windows open. The estimated inhaled doses over the 2.7 km route were 6.83 μg for walking, 2.78 μg for cycling, 1.28 μg for light-rail train, 1.24 μg for driving with windows open, 1.23 μg for bus, and 0.32 μg for driving with windows closed. Similarly, the exposure rates were highest for cycling (18.0 μg/hr) and walking (16.8 μg/hr), and lowest for driving with windows closed (3.7 μg/hr). Our findings support previous studies showing that active commuters receive a greater PM_2.5_ dose and have higher rates of exposure than commuters using automobiles or public transportation. Our findings also support previous studies showing that driving with windows closed is protective against traffic-related PM_2.5_ exposure.

## Introduction

Exposure to fine particulate matter (PM_2.5_) in ambient air is associated with multiple adverse health outcomes in adults and children [1]. In urban areas, motor vehicle exhaust contributes significantly to PM_2.5_ air pollution [2, 3]. There is extensive spatial and temporal variation in intra-urban air pollution exposures [4], with PM_2.5_ often occurring at higher concentrations near major roadways due to gasoline and diesel engine exhaust [5]. Thus, for commuters, traffic-related air pollution can significantly contribute to daily PM_2.5_ exposures, especially on arterial roads during high-traffic “rush hour” time windows [6]. American commuters spend approximately 26 minutes commuting one direction to work each day [7], making long-term and acute health effects from exposure a concern [8, 9].

One strategy to reduce daily PM_2.5_ exposure is to encourage a shift in modal transportation toward increased use of public transit and active commuting (walking and cycling). However, to date there are relatively few studies on PM_2.5_ exposures by type of commuting in the United States. Some studies show higher PM_2.5_ exposures for cyclists [10] and walkers [11, 12] compared with automobile commuters, whereas others report no difference [13] or lower exposures for active commuters compared to those using automobiles or public transit [14, 15]. Complicating the issue, walking and cycling take longer and increase individuals’ breathing rates, potentially exposing active commuters to higher inhaled doses of PM_2.5_ compared to automobile or public transit [10, 12, 16, 17]. There is significant variability in automobile cabin exposure depending on window position (open or closed) and whether the car’s internal air circulation is turned off, which allows outdoor air to be entrained into the vehicle [18]. Thus, additional research is needed to better characterize PM_2.5_ exposure by transportation mode.

Understanding inter-modal differences in commuters’ PM_2.5_ exposures is difficult for a number of reasons. Zuurbier et al. (2010) note that in most previous studies, particulate exposures were not measured simultaneously by mode of commuting [16]. This is problematic due to spatial and temporal fluctuations in background air pollution levels (e.g. due to a geographic depression, or commuting-time vs. night-time levels) [19, 20]. Some studies have tried to adjust for this by making comparisons with data from fixed-site monitors (FSM) [10, 14, 21, 22]. However, there can be considerable variability in PM_2.5_ measures based on differences in sampling equipment [23], making direct comparisons between FSMs and personal exposure monitors difficult [16]. There is also considerable variability associated with commuting microenvironments. This variability includes active commuters’ flexibility in choosing a wider variety of routes. To control for this variation, previous studies have used prescribed commuting routes. However, many of these routes had limited comparison groups (e.g. walking vs. driving) [10, 12], or only looked at one particular mode of transportation (e.g. bicycles) [22, 24]. Another challenge has been that all transport modes evaluated in some studies were unable to use the exact same route (i.e. subway vs. car) [14].

In this study, we examined personal exposures to PM_2.5_ for six different commuting types (bicycle, walking, driving with windows open and closed, bus, and light-rail train) on a single prescribed arterial urban route during peak “rush hour” times. To control for variation in background PM_2.5_ we used identical instruments for both personal and ambient monitoring. Finally, we estimated the PM_2.5_ inhaled dose and rate of exposure for each commuting mode based on previously published minute ventilation rates [16, 25, 26].

## Materials and methods

### Setting

**Fig 1:**
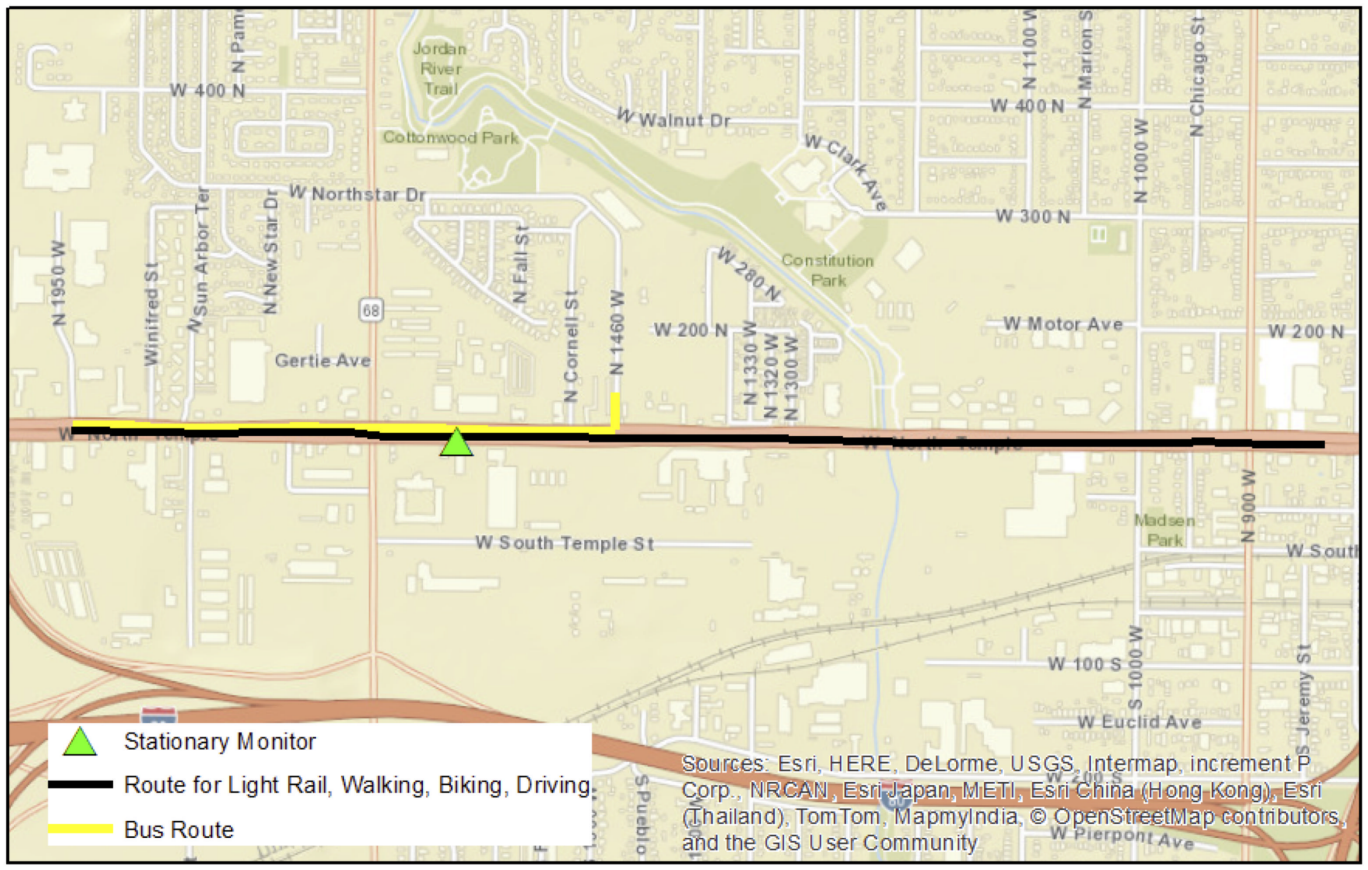
Designated commuting route for all forms of transportation and location of the stationary monitor.

Salt Lake City (SLC) is the capital and largest municipality in Utah, with a population of 192,672 according to the 2015 United States census [27]. Population density in SLC is 657.5 people/km^2^ (1,703 people/mi^2^) [28]. Air pollution samples were collected on a 2.7 km (1.68 mi) section of road located between 600 W and 1950 W, N. Temple in Salt Lake City, Utah. We chose this location because it serves as a main transportation corridor leading into the downtown SLC area. This route provides access to multiple forms of transportation, including two traffic lanes each for eastbound and westbound automobiles, bicycle lanes and sidewalks on both sides of the roadway, diesel bus routes, and an electric light rail system located between eastbound and westbound lanes. This route is located on the west side of SLC, where the terrain is flat. This roadway is zoned for commercial business, and includes restaurants, shops, office buildings, banks, and grocery stores. Several blocks on the north side of the road border on the Utah State fairgrounds. Residential areas are located within one block of the roadway at some locations (Fig 1).

### Personal monitoring

We measured breathing zone PM_2.5_ concentrations on 15 study personnel using TSI SidePak AM510 personal exposure monitors (TSI, Inc., Shoreview, MN, USA) as they travelled the 2.7 km (1.68 mi) route via walking, biking, riding the bus, riding the light rail system, driving with windows open, and driving with windows closed. Study personnel were university faculty (n = 3) and students (n = 12), all of which were in good physical health. For both automobiles, the internal circulation system was turned on during all study periods. All study personnel were non-smokers. Data collection occurred from Aug 8^th^–Aug 11^th^, 2016. Air pollution monitoring was conducted from approximately 8:00–10:00 AM, and again from 4:00–6:00 PM during peak commuting times. Samples were collected simultaneously for all modes of transportation beginning at the same time. At times, study workers were shifted to less efficient modes in order to provide enough overall sampling time per mode. However, individual data collection events occurred more expeditiously for some modes of transportation (e.g. light rail and driving) than for others. For the purposes of this study, a data collection event was defined as the study worker initiating data logging on the SidePak, travelling the length of the route in one direction using their designated mode of transportation, and then discontinuing data logging. Once the data collection event was completed, the study worker crossed to the other side of the road and repeated this process. For study workers riding the light rail, SidePak data logging was initiated approximately 5 minutes before arrival of the train, and was stopped upon exiting the train. The worker then reset the SidePak and rode the next available train in the opposite direction. There were no busses that ran the entire length of the study roadway, so we were obliged to use a route that covered approximately 44% (1.19 km) of the entire study route on the north side of the road only (Fig 1).

SidePak AM510s (n = 9) were set to record PM_2.5_ measures every 1-second during sampling. Each night prior to the next day’s sampling, we reset the instruments’ internal clocks to the lab computer date/time, cleaned and greased the impaction plates, recorded post-sampling air pump flow rates (post-calibration), calibrated pump flow rates to 1.7 L/min (pre-calibration for the next day) using a Defender 510 volumetric flow calibrator (Mesa Labs, Butler, NJ, USA), zeroed the 670 nm laser using an in-line HEPA filter, and charged the batteries. All pre- and post-calibrations were within ±5% of the 1.7 L/min target flow rate. All of the SidePak AM510 instruments were calibrated by the manufacturer to ISO 12103–1 A1 test dust (Arizona road dust) prior to using them in this study.

The inlet to the SidePak AM510 was attached to the collar or shoulder of the study worker’s shirt so it was within the person’s breathing zone. For the purposes of this study, we defined the breathing zone as the sphere of air located within 25 cm (10 in) of the individual’s nostrils [29]. To control for differences in particle release by type of clothing, all study personnel who wore a personal monitor wore a standardized high visibility, 100% polyester shirt over the top of their regular clothing. Previous studies have used vests made from nylon or 50:50 cotton:polyester blends [30, 31]. There is little data on fabric particle release, so we chose 100% polyester based on the assumption that synthetic fabrics emit less dander than fabrics made with natural fibers, such as cotton.

### Stationary monitoring

Ambient PM_2.5_ was measured with one stationary SidePak AM510 instrument placed on the south side of the road (Fig 1). The inlet for the SidePak stationary monitor was located approximately 0.9 m (3 ft.) off the ground on a stand, and placed approximately half way between the edge of the road and the sidewalk. Stationary monitoring was performed whenever personal breathing zone samples were being collected. The stationary monitor was calibrated and maintained as described for the personal monitors.

### Instrument validation

Two university-owned Toyota Sienna mini-vans, 2013 and 2016 models, were used in this study for monitoring breathing zone exposures for driving with windows open and driving with windows closed. For driving with windows closed, van air conditioning was used with the air recirculating in the cabin (not drawing outside air into the vehicle). Both vehicles used the same model of cabin air filter (Toyota part number 87139-YZZ20), which had an atmospheric dust efficiency rating of 0.3 ~ 0.5 μm: 20±10% at 240m^3^/h. The same cabin air filter was used to remove particulate matter coming into the vehicle from outside and when recirculating air inside the vehicle. University maintenance records showed the air filters had not been replaced, and thus were original to the van. To verify that differences in personal monitoring between the two vans were not due to air pollution produced by either van leaking into the cabin, we conducted personal monitoring simultaneously in both vans while driving the length of the route with windows closed. We first evaluated whether the 1-second measurements were autocorrelated using the Durbin Watson test. We found significant autocorrelation (p = 0.023), which was expected due to the short, 1-second time interval used on the SidePaks. Thus, to compare means between the two vans, we performed a t-test taking into account serial correlation in the data. Our results showed a statistically significant difference between the two vans (p = 0.029). Means and standard errors were 5.2 μg/m^3^ (SE = 0.17) and 4.9 μg/m^3^ (SE = 0.13) for the 2016 and 2013 models, respectively. Although the difference between the two vans was statistically significant, we believe the 0.3 μg/m^3^ difference was not necessarily clinically significant from an overall exposure standpoint. JMP version 12.0 (SAS Institute Inc., Cary, NC, USA) was used for this analysis.

Relative humidity (RH) greater than 50% can cause hygroscopic enlargement of particles less than 2.5 μm, resulting in the SidePak overestimating the concentration of PM_2.5_ [11]. Ambient hourly RH measurements over the study period were obtained from the Hawthorne monitoring station operated by the Utah Department of Environmental Quality, Division of Air Quality (DAQ). The Hawthorne monitoring station, the closest DAQ monitor, is located 6.79 km from the study route. For the morning and afternoon commutes, the average of the DAQ hourly means was 30.1% (range = 15.9–48.1%) and 18.6% (range = 9.0–38.7%), respectively. Based on the low ambient RH we expected inappreciable hygroscopic particle growth, and thus made no corrections to the SidePak measurements.

### Data analysis

We cleaned the data by matching each data point of commuter (personal monitoring) with the particulate levels measured by the stationary monitor by date and second. The detection limit of the SidePak recorded by TSI is 0.001mg/m^3^ with a zero stability of ±0.001 mg/m^3^ over 24 hours. In order to account for this known limit, readings below 0.001 mg/m^3^ were recoded as 0.0007 mg/m^3^, which is the detection limit divided by the square root of two. This allowed us to use readings where the SidePak read 0 mg/m^3^ due to the monitor’s inability to detect particle counts that low (the particle count is never truly zero), and proceed with analysis at those time points. We then calculated the ratio of each commuting type to the background levels recorded at each second. The ratios were averaged and reported along with standard errors for overall particulate exposures by commuting type and by commuting type and time of day (morning or afternoon rush hour). Statistical differences between groups were calculated using linear regression within R: *A Language and Environment for Statistical Computing* (v 3.3.2) [32].

To calculate the inhaled dose of PM_2.5_ per type of commuting, we used the averages of minute ventilation rates based off of different studies. Car transportation, with both windows opened and closed, had a minute ventilation rate of 11.8 L/min [16]. Light rail and bus transportation were considered similar enough to use the same minute ventilation rate: 12.7 L/min [25]. Cycling and walking had higher minute ventilation rates due to the physical nature of this commuting method. Walking had the second highest minute ventilation rate of 22.8 L/min [26]. Cycling had the highest at 23.5 L/min [16]. The inhaled dose (μg PM_2.5_ per trip) was then calculated by multiplying the mean PM_2.5_ concentration for each commuting type by the minute ventilation rate (L/min) and then by multiplying by the conversion factor 1m^3^/1000 L and by the average number of minutes per trip [33]. Exposure rates were calculated by dividing the inhaled dose (μg) by the average trip time (hrs).

## Results

We recorded real-time PM_2.5_ concentrations for six different commuting types and one stationary monitor in downtown Salt Lake City, Utah. We collected between 4524 (bus) and 39,725 (walking) data points for each type of commuting. The disparity in the numbers of points is due to inherent differences in commuting. The bus comes infrequently, whereas walkers were able to record values continuously. It should also be noted that due to a technical malfunction, data for driving with windows closed was only recorded on 8/8/16 and 8/11/16. While this malfunction was unfortunate, the particulate matter for driving windows closed was very consistent, with a standard deviation in the raw data of only 4.6 ug/m^3^. Overall standard deviations for the other modes of transportation varied between 9.9 ug/m^3^ (bike) and 17.2 ug/m^3^ (light rail). The standard deviations for driving with windows open, bus and walking were 11.4 ug/m^3^, 11.6 ug/m^3^ and 12.6 ug/m^3^, respectively. It is therefore unlikely that the comparative results were strongly affected by the omission of driving windows closed on the other days.

Using the clean data matched by second to the ambient monitoring station, the average concentration of PM_2.5_ experienced by the different commuting types was between 5.21 ug/m^3^ (driving with windows closed) and 15.21 ug/m^3^ (driving with windows open). Mean exposures were 12.49 ug/m^3^ for light rail, 12.21 ug/m^3^ for walking, 12.62 ug/m^3^ for biking and 13.03 ug/m^3^ for taking the bus. While these averages are of interest, they may be differentially influenced by background pollution levels. Therefore, we focus the reporting of results below on the ratio of personal exposure by commuting type to background levels at each recorded second.

**Fig 2:**
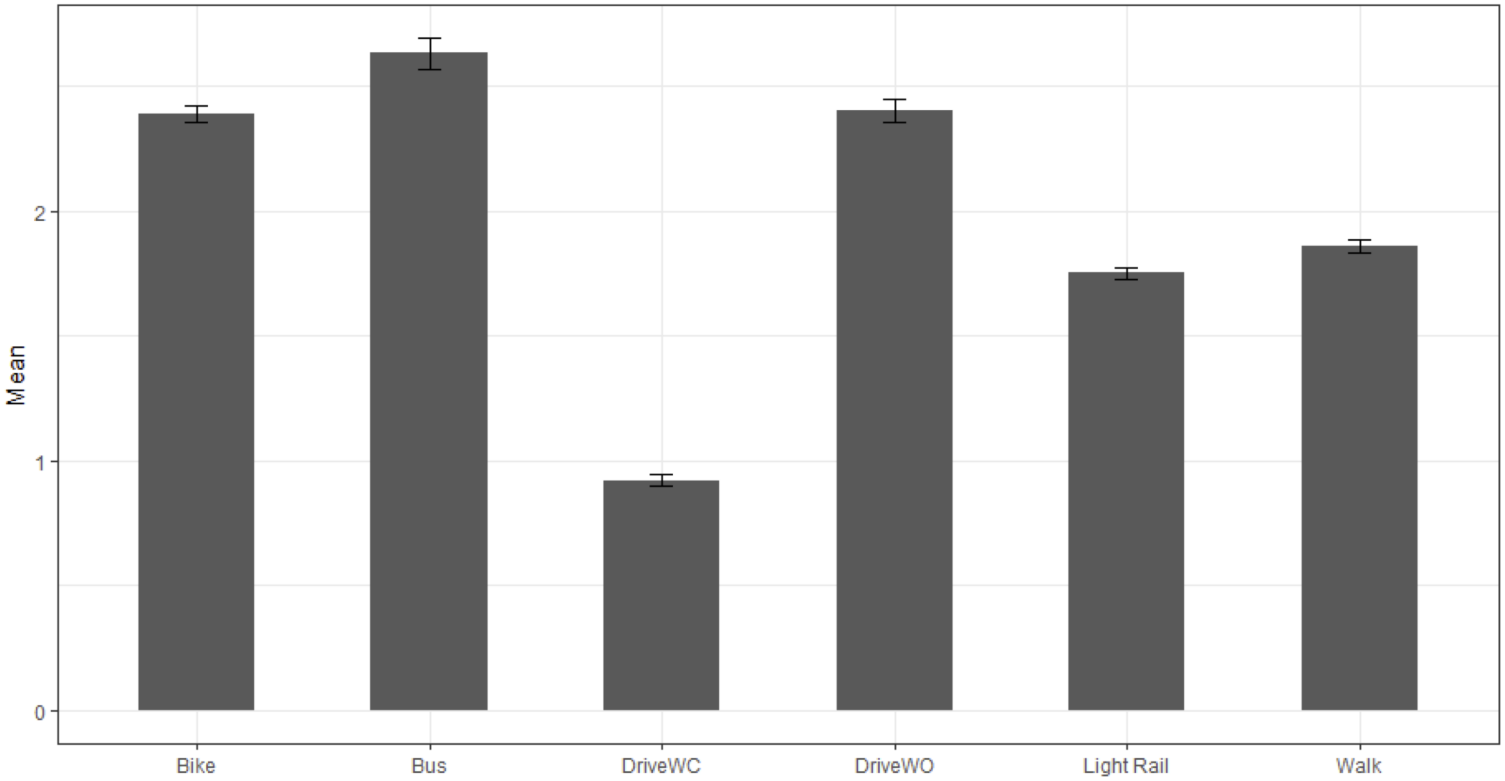
Mean ratios of PM_2.5_ between personal and stationary monitor values by commuting type. Bars represent standard errors.

**Fig 3:**
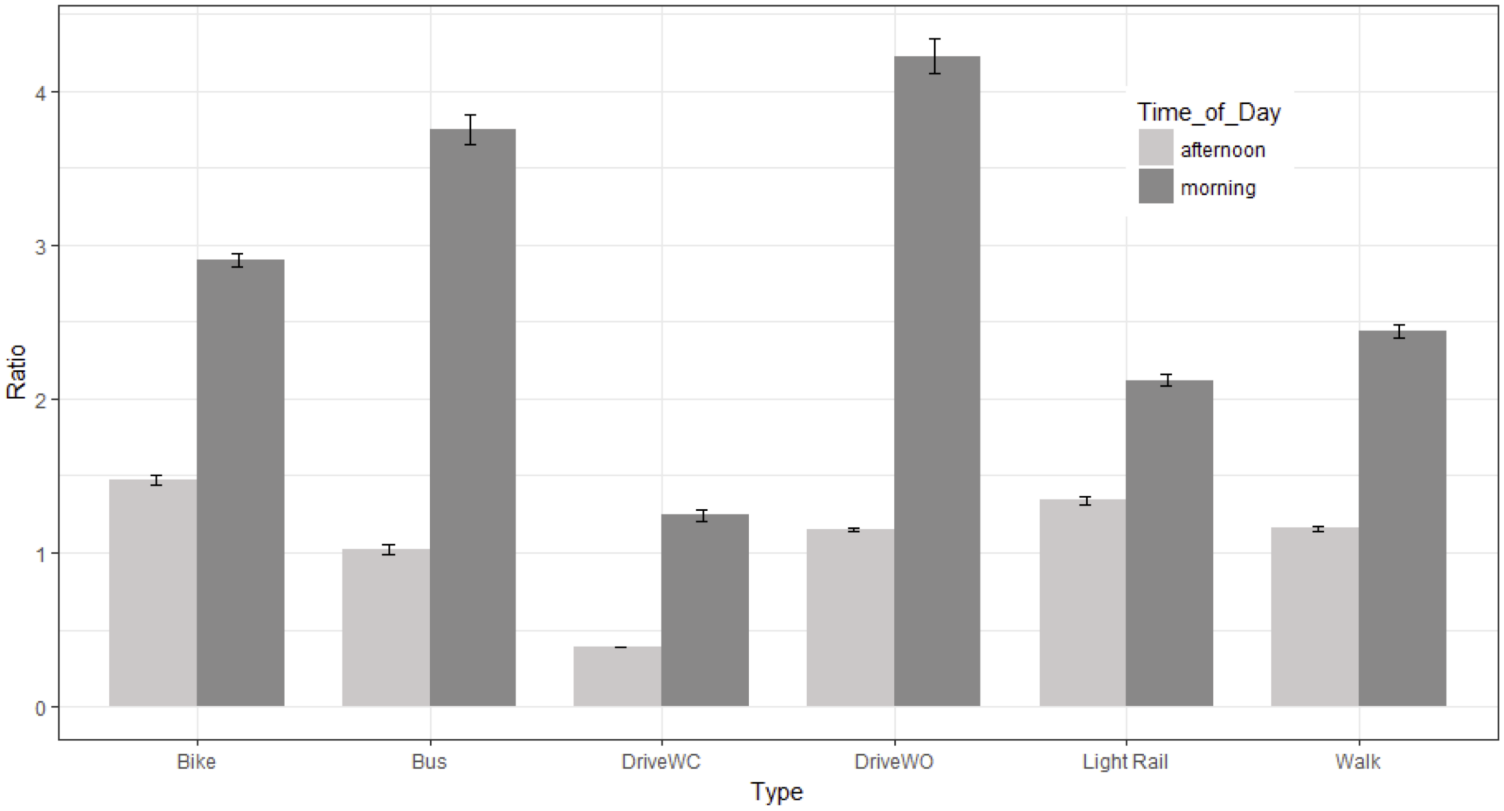
Mean ratios of PM_2.5_ between personal and stationary monitor values by commuting type and time of day. Bars represent standard errors.

The number of data points (seconds), means, medians, variances and standard errors of the ratios of PM_2.5_ by commuting type and background monitor are shown in Table 1. Data are reported by type, date and time of day. The overall average ratio of particulate matter to background monitor was highest for commuting by bus (2.63). The lowest was for driving with the windows closed (0.93). Driving with windows closed was the only method of commuting that had an average ratio of less than 1 compared to the background monitor. The means were influenced by widely different variances by day, time and commuting type, as mentioned. For example, on the morning of 8/11, the walker experienced some very high particulate matter levels, with a variance of 163.21. The median values for all commuting types are therefore also informative, and ranged from 0.45 (driving windows closed) to 1.33 (bus). Ratio means and standard errors are shown in Figs 2 and 3 for overall exposure and exposure by time of day (morning or afternoon).

**Table 1:** Descriptive statistics of breathing zone/background PM_2.5_ ratios by mode of commuting.

Type	Time of Day	Date	Data Points (N)^1^	Mean	Median	Variance	StdErr	25% Quartile	75% Quartile
Bike	morning	8/8/16	811	1.20	1.09	0.43	0.02	0.93	1.33
Bike	afternoon	8/8/16	2642	0.96	0.89	0.21	0.01	0.76	1.06
Bike	morning	8/9/16	2495	2.41	1.75	8.93	0.06	1.20	2.67
Bike	afternoon	8/9/16	1877	1.11	1.00	0.40	0.01	0.80	1.27
Bike	morning	8/10/16	5205	1.97	1.14	12.43	0.05	0.74	1.83
Bike	afternoon	8/10/16	1076	2.59	1.50	21.22	0.14	1.00	2.67
Bike	morning	8/11/16	2657	5.70	3.00	51.91	0.14	1.60	7.25
Bike	afternoon	8/11/16	646	2.77	1.50	24.21	0.19	1.00	2.67
		**Total**	**17409**	**2.39**	**1.22**	**17.50**	**0.03**	**0.85**	**2.14**
		% BDL: 0.16							
Bus	afternoon	8/8/16	568	0.95	0.80	0.30	0.02	0.63	1.14
Bus	morning	8/9/16	655	2.66	2.17	3.72	0.08	1.25	3.63
Bus	afternoon	8/9/16	377	1.17	1.00	7.75	0.14	0.73	1.20
Bus	morning	8/10/16	1234	3.35	1.80	26.17	0.15	1.13	3.00
Bus	afternoon	8/10/16	195	1.40	1.13	1.38	0.08	0.88	1.44
Bus	morning	8/11/16	782	5.30	3.33	35.35	0.21	2.00	6.67
Bus	afternoon	8/11/16	713	0.89	0.83	0.20	0.02	0.63	1.09
		**Total**	**4524**	**2.63**	**1.33**	**17.00**	**0.06**	**0.85**	**2.60**
		% BDL: 0.12							
Drive WO^2^	morning	8/8/16	1332	1.24	1.10	0.74	0.02	0.93	1.39
Drive WO	afternoon	8/8/16	2415	0.98	0.90	0.33	0.01	0.76	1.08
Drive WO	morning	8/9/16	1618	2.97	2.20	15.13	0.10	1.40	3.33
Drive WO	afternoon	8/9/16	1963	1.20	1.09	0.44	0.02	0.92	1.33
Drive WO	morning	8/11/16	1969	7.28	4.00	132.14	0.26	2.07	9.00
Drive WO	afternoon	8/11/16	2756	1.25	1.10	1.22	0.02	0.86	1.40
		**Total**	**12053**	**2.40**	**1.19**	**29.12**	**0.05**	**0.90**	**1.90**
		% BDL: 1.17							
Drive WC^2^	morning	8/8/16	946	0.69	0.50	0.35	0.02	0.31	1.00
Drive WC	afternoon	8/8/16	529	0.43	0.41	0.02	0.01	0.35	0.47
Drive WC	morning	8/11/16	2142	1.50	0.80	5.23	0.05	0.33	1.79
Drive WC	afternoon	8/11/16	1325	0.38	0.36	0.02	0.00	0.27	0.45
		**Total**	**4942**	**0.93**	**0.45**	**2.60**	**0.02**	**0.31**	**1.00**
		% BDL: 0.20							
Light Rail	morning	8/8/16	850	1.82	1.79	0.30	0.02	1.50	2.09
Light Rail	afternoon	8/8/16	3209	1.26	1.20	0.30	0.01	1.00	1.42
Light Rail	morning	8/9/16	2636	2.52	1.67	22.61	0.09	1.00	2.75
Light Rail	afternoon	8/9/16	1737	1.34	1.25	0.41	0.02	1.00	1.55
Light Rail	morning	8/10/16	3489	1.44	0.86	5.39	0.04	0.55	1.40
Light Rail	afternoon	8/10/16	1935	2.08	1.00	27.30	0.12	0.64	1.63
Light Rail	morning	8/11/16	2933	2.68	1.43	20.63	0.08	0.67	3.00
Light Rail	afternoon	8/11/16	2045	0.77	0.64	0.38	0.01	0.50	0.86
		**Total**	**18834**	**1.75**	**1.14**	**10.73**	**0.02**	**0.71**	**1.72**
		% BDL: 0.54							
Walk	morning	8/8/16	4262	1.13	1.00	0.80	0.01	0.77	1.23
Walk	afternoon	8/8/16	4236	0.78	0.71	0.23	0.01	0.59	0.87
Walk	morning	8/9/16	3321	2.19	1.50	7.87	0.05	1.00	2.50
Walk	afternoon	8/9/16	3057	0.91	0.80	0.49	0.01	0.64	1.00
Walk	morning	8/10/16	8691	1.70	1.33	3.65	0.02	0.83	2.00
Walk	afternoon	8/10/16	6454	1.67	1.10	7.67	0.03	0.75	1.70
Walk	morning	8/11/16	5444	4.79	2.50	163.21	0.17	1.25	5.12
Walk	afternoon	8/11/16	4260	0.94	0.70	0.95	0.01	0.55	1.00
		**Total**	**39725**	**1.86**	**1.00**	**26.86**	**0.03**	**0.70**	**1.75**
		% BDL: 0.10							

^1^ SidePak AM510 personal exposure monitors (TSI, Inc., Shoreview, MN, USA) were set to record measurements every second, and therefore the total number of minutes for each mode of commuting can be derived by dividing the number of data points by 60.

^2^ Drive WO and Drive WC = driving with windows open windows closed, respectively.

The linear regression analysis showed that all commuting types were statistically significantly different from the reference type, driving windows closed (p < 0.001). Heteroscedasticity was not present in the residuals as determined by the Breush-Pagan test.

As shown in Table 2, the inhaled dose (μg) was highest for active commuters. Walkers and cyclists’ inhaled doses were 21.3 and 8.7 times higher, respectively, compared with driving with windows closed over the same 2.7 km route. Inhaled doses for driving with windows open and light rail were 3.9 and 4.0 times higher, respectively, compared with driving with windows closed. Although the bus did not travel the entire route, we estimated the inhaled dose (1.23 μg) for bus commuters, based on a 7.45 min travel time, to be 3.8 times higher than driving with windows closed. When calculated by μg/hr of commuting, active commuting by bike and walking resulted in PM_2.5_ exposure rates that were 4.8 and 4.5 times higher, respectively, than driving with windows closed. This was due largely to the elevated minute ventilation rates associated with active commuting and the lower PM_2.5_ concentration inside cars with windows closed. Exposure rates for driving with windows open, light rail, and bus were 2.9, 2.6, and 2.7 times higher, respectively, than driving with windows closed.

**Table 2:** Inhaled dose and exposure rates of fine particulate matter by type of commuting.

Type of Commuting	N Trips	Avg Trip Time (minutes (sd))	PM_2.5_ Concentration (μg/m^3^)	Minute Ventilation Rate (L/Min)	Inhaled Dose (μg)	Exposure Rate (μg/hr)
Drive WC	16	5.15 (3.91)	5.21	11.8	0.32	3.7
Drive WO	29	6.93 (2.93)	15.21	11.8	1.24	10.8
Light Rail	39	8.05 (5.28)	12.49	12.7	1.28	9.6
Walk	27	24.52 (4.68)	12.21	22.8	6.83	16.8
Bike	31	9.36 (2.34)	12.62	23.5	2.78	18.0
Bus^1^	23	3.28 (1.23)	13.03	12.7	0.54	10.2

^1^ The distance for bus commuting was only 44% of the entire 2.7 km (1.68 mi) route, thus resulting in a lower inhaled dose of fine particulate matter. Had the bus traveled the entire route, we estimated the trip time to be 7.45 min, resulting in an inhaled dose of 1.23 μg.

## Discussion

In this study, we examined fine particulate matter exposure for six different commuting types simultaneously on a single prescribed urban route during “rush hour” times. Breathing zone PM_2.5_ concentrations were highest for driving with windows open and bus travel, and lowest for driving with windows closed. However, other factors such as travel time, mode of commuting, and minute ventilation rates can significantly influence the rate of exposure and overall inhaled dose of particulate matter [10, 16]. Considering these factors, our findings support previous studies that estimate the inhaled dose of PM_2.5_ for active commuters to be 2–7 times greater than for automobile and public transit commuters over comparable routes [12, 16]. Similarly, McNabola et al. (2008) estimated that cyclists and pedestrians in their study would experience greater PM_2.5_ lung deposition than car commuters, largely due to increased respiration rates [17]. By comparison, the estimated inhaled doses of PM_2.5_ for cyclists and walkers in our study were approximately 9 and 21 times greater, respectively, than driving with windows closed over the same 2.7 km route.

To date, most studies compare exposures across modes of transportation in terms of breathing zone concentrations (μg/m^3^); however, inhaled dose (μg) and exposure rate (μg/hr) may be better measures for understanding health outcomes associated with commuting. Several previous studies show no difference or lower PM_2.5_ concentrations for cyclists and walkers than for automobile commuters [13–15], but these studies did not account for the higher respiration rate for active commuters. In our study, driving with windows open and riding the bus exposed commuters to the highest breathing zone concentrations, but resulted in relatively low inhaled doses and exposure rates compared to active commuters. Zuurbier (2010) found similar results, where the highest median PM_2.5_, PM_10_, and soot concentrations were among automobile and bus commuters, but the highest exposure rates were found among high-traffic cyclists [16]. In their study, PM_2.5_ exposures during cycling occurred at a rate of 100.4 μg/hr, compared to 51.9 μg/hr for diesel car [16]. By comparison, exposure rates in our study for cycling, walking, and driving with windows closed were 18.0, 16.8, and 3.7 μg/hr, respectively. Commuting accounts for 6–10% of the day for many workers, but may contribute up to 12% of daily PM_2.5_ exposure [21, 34, 35]. Based on our results, we suggest the daily PM_2.5_ contribution attributable to commuting for cyclists and walkers may be 4–5 times higher than among automobile users. There is currently little data on the long-term health effects of increased air pollution exposure among active commuters, warranting additional research in this area [34].

We anticipated driving with windows closed with the car’s air conditioning with air recirculating on in the cabin (not drawing outside air into the vehicle) to result in the lowest exposure rates. Closing the vehicle windows and turning on the air recirculation system creates a mostly-closed, protective bubble around the automobile’s occupants. However, Knibbs, de Dear, and Atkinson (2009) described that even vehicles with closed windows experience some air flow through “leaks” [36]. A variety of sources can cause leaks, but they seem to be increasingly common in older vehicles, those with poor quality seals around doors and windows, poor quality filtration systems, and leaks through the bulkhead. Based on this, newer vehicles, like those used in this study, seem to allow the least outside air to enter the cabin (i.e. be the least “leaky”), and thus may provide the greatest protection against traffic-related air pollution. Conversly, Ott, Klepeis, and Switzer (2008) explained that opening a window significantly increases exchange between cabin and outdoor air (e.g. opening a window just 3-inches increased the air exchanges per hour by 8–16 times) [18]. It should be noted, that variation in protection offered by closed windows is dependent on the vehicle’s air filter, the traffic congestion, and air circulation settings [37]. From our study, we see that driving with windows closed with the automobile’s air conditioning with air recirculating on in the cabin (not drawing outside air into the vehicle), despite “leaks,” protects the commuter from outdoor air pollution, whereas driving with windows open significantly increases one’s exposure.

Our findings support Briggs et al. (2008) who showed that walking exposed commuters to 2.2 times higher mean PM_2.5_ concentrations compared to driving with the car windows closed. In their study, the car’s air conditioner was turned off, and the ventilation system was set to a “moderate” level [12]. Our results were very similar. We found that walking and cycling exposed commuters to 2.3 and 2.4 times higher mean PM_2.5_ concentrations, respectively, compared to driving with windows closed. In our study, the car’s air conditioning system was used with the air recirculating on in the cabin (not drawing outside air into the vehicle). However, other studies show in-car exposures can be higher than for cyclists and walkers [16, 17]. In Zuurbier’s (2010) study [16], car windows were closed and the air conditioner was used on a “moderate” level; and in McNabola’s (2008) study [17], windows and vents were closed and the air conditioner was not used. It is not clear from each study’s methods whether the internal air circulation system was turned on or off. We suggest this may be an important factor that contributes to the protection provided by closed-window driving. Future studies are needed to compare ventilation system configurations for driving in high pollution environments to determine the optimal settings to reduce exposures.

A combination of leaks when windows are closed and infiltration of outdoor air when windows and doors are open likely explain the high level of air pollution in busses. Sabin et al. (2005) describe that busses, especially older ones, can self-pollute (i.e. particles enter the bus even though the windows are closed), and passenger exchange can exacerbate this effect [38]. Hammond, Jones, and Lalor (2007) explain that particle exposure on busses is also affected by bus idling and ventilation in that [39]. Data collection for our study occurred during August, which is usually the hottest month of the year in Utah. As is typical during hot summer months, the busses on the route used in this study had the air-conditioning system turned on, but we did not systematically measure if windows were opened or closed. Given the previously reported findings, it is likely that the high PM_2.5_ concentrations in busses in our study were due to self-pollution via leaks and air exchange between the cabin and outdoor air when the bus doors were opened during stops or from windows being opened.

We found considerable variation in PM_2.5_ levels among all transportation modes with the exception of driving with windows closed. The most pronounced differences were seen for driving with windows open and walking, which had more than 10 times greater average variation than driving with windows closed. The high variation in exposure for driving with windows open may be explained by greater PM_2.5_ concentrations entering the vehicle while idling at stoplights and lower concentrations experienced while driving. The variability experienced by walkers is likely due to microenvironments along the walking path [13]. Over a comparable route, a walker would have more time to experience both extremes of very low and very high exposure concentrations. Variation in PM_2.5_ measures also fluctuated significantly by day and time of day. For instance, the highest average variation in exposure for all modes (except light rail) occurred on the morning of August 11. Similarly, the lowest average variation for all modes (except bus) occurred in the afternoon on August 8 (Table 1). Traffic density and wind speed & direction influence PM_2.5_ exposures [16, 40], and may account for the large variations seen across days/times in this study. Unfortunately we did not measure these variables, and can only speculate as to their effect. Closing car windows and turning on the internal air circulation system appears to create a protective “bubble” around the vehicle occupants during commuting, resulting in significantly lower variation in PM_2.5_ exposures.

There are important practical implications from this research. First, studies suggest the health benefits of active commuting outweigh the risks of higher air pollution exposures [10, 16, 34]. These benefits include reduced risk for several chronic diseases such as obesity, cardiovascular disease, and cancer [41]. However, there is little data on commuters’ perceptions about air pollution exposure and how these perceptions may influence their commute in terms of route, time of day, and mode of transportation. Understanding these perceptions may help guide future educational efforts aimed at reducing active commuters’ air pollution exposures. Second, public transportation (bus and light rail) disproportionately exposed commuters to about four times greater inhaled doses of PM_2.5_ over the same route compared with automobile commuters who drove with windows closed. This may pose a greater lifetime risk of cardiopulmonary mortality and lung cancer to minority commuters, who tend to use public transportation more often than Caucasians; likewise, roughly 65% of public transportation users have a household income less than the national median household income [42, 43]. Third, driving with windows open exposed commuters to the highest concentration of pollutants. As with active commuting, educational campaigns directed at automobile commuters may help lower exposures by increasing their knowledge and self-efficacy for optimizing their vehicle’s ventilation system to reduce PM_2.5_ concentrations in the car. Finally, safety and infrastructure are commonly evaluated in promoting active transportation; however, air pollution exposures should be another important consideration in city planning when selecting routes for active commuters.

This study was limited to examining commuter data on one roadway in a single metropolitan city during summer, and therefore may not be generalizable to other locations or seasons. We were unable to account for the large variability in PM_2.5_ measures by mode of commuting, day, and time. In retrospect, measuring traffic density and the types of vehicles using the road may have helped in this regard. We used real-time particle counters to measure breathing zone concentrations, which did not allow for chemical speciation of PM_2.5_. Likewise, we only measured PM_2.5_ not ultrafine or coarse PM, or other gaseous pollutants (e.g. ozone). The SidePak AM510 instruments measured PM_2.5_ concentrations indirectly using 90° light scattering based on ISO 12103–1 A1 calibration test dust. Thus, actual ambient PM_2.5_ concentrations may have differed from our readings due to differences in particle size, shape, refractive index, and chemical composition; however, we assume that any measurement errors were systematic and roughly proportionate among all modes of commuting. Finally, although we did re-zero the SidePak instruments each night, we did not check for zero drift immediately before or after sampling. It is possible that temperature differences between the laboratory and sampling site location caused the SidePaks to read higher in the afternoon as temperatures increased.

Studies suggest the health effects associated with PM_2.5_ exposure in adults and children may depend on its chemical makeup [44–47]. Fondelli et al. (2008) found differences in fine particle sulfur concentrations between busses and taxis [21], suggesting additional research is warranted to understand how PM_2.5_ constituents vary by mode of commuting. This may provide additional valuable information to help direct future public health policy recommendations specific to commuting. This study was also limited in that we did not measure minute ventilation rates, but used values reported in previous studies. This may have resulted in under or over-estimation of actual inhaled dose and exposure rates due to differences in the fitness levels of study participants, speed of travel, weather conditions, and grade (flat vs sloped terrain) between this and other studies. In some settings, active transportation users choose different routes than those using motorized transportation. This study fixed route choice in order to make comparisons between transportation modes. Strengths of this study include the use of an identical background monitor at the sampling location, measurement of six commuting modes simultaneously on a common route, and controlling for dander released from clothing by using the same over-shirt for all data collection personnel.

In conclusion, our data suggests that active commuting (walking and cycling), when compared with other modes of transportation over comparable distances and routes, results in significantly higher PM_2.5_ doses and exposure rates. The higher exposure doses and rates estimated in our study were largely a function of increased respiration rate and time of travel for active commuters. Furthermore, driving with windows closed with the car’s internal air circulation system turned on was protective, resulting in the lowest PM_2.5_ breathing zone concentration, inhaled dose, and exposure rate. Future research should evaluate differences in fine particle constituents based on mode of commuting, including in congested conditions. These comparisons should also include relevant biological measurements. Furthermore, we recommend future studies to evaluate public awareness of how transportation choices influence commuters’ daily exposures.
